# Gout with auricular tophi following anti-tuberculosis treatment: a case report

**DOI:** 10.1186/1756-0500-6-480

**Published:** 2013-11-21

**Authors:** Hsin-Jen Chang, Pa-Chun Wang, Ying-Chieh Hsu, Shih-Hung Huang

**Affiliations:** 1 Department of Otolaryngology, Head and Neck Surgery, Cathay General Hospital, Taipei, Taiwan; 2 School of Medicine, Fu Jen Catholic University, Taipei, Taiwan; 3 School of Public Health, China Medical University, Taichung, Taiwan; 4 Department of Pathology, Cathay General Hospital, Taipei, Taiwan

**Keywords:** Tophus, Auricle

## Abstract

**Background:**

Auricular tophi are firm deposits of monosodium urate in crystal form, which may slowly develop in subcutaneous tissue of the ear. Ear is not usual locations for gout tophi, but when this growth does occur, helix and the antihelix are common sites.

**Case presentation:**

We present a 64-year-old man who had multiple painless nodules over bilateral helix. An excisional biopsy was performed. Hematoxylin-eosin staining of biopsy specimens revealed a proteinaceous matrix that surrounded dissolved crystals, consistent with gout tophi. Bilateral auricular tophi are not common and may resemble a number of other diseases including squamous cell carcinomas, Kaposi’s sarcoma, epidermal and dermoid cysts, rheumatoid nodules. Biopsy should be performed to rule out malignancy.

**Conclusions:**

Tophi of the auricle are usually asymptomatic but can become inflamed and occasionally ulcerate through the overlying skin. Chronic tophaceous gout is treated with dietary control and medication. Surgical excision is performed under local anesthetic if symptoms progression or cosmetically deformity is concerned.

## Background

Auricular tophi are firm deposits of monosodium urate in crystal form, which may slowly develop in subcutaneous tissue. Gout tophi occurs around auricular area is not common. Common locations for this growth on the ear are the helix and the antihelix. We present a 64-year-old man who had multiple painless nodules over bilateral helix after he received anti-tuberculosis therapy. Auricular tophi are not common and may resemble a number of other diseases including squamous cell carcinomas, Kaposi’s sarcoma, epidermal and dermoid cysts, rheumatoid nodules. Biopsy should be performed to rule out malignancy.

## Case presentation

A 64-year-old man was seen with a 6-month history of multiple bilateral auricular nodules. He also had a history of pulmonary tuberculosis and had been receiving antituberculosis therapy since December 2009. The protocol consisted of 4 drugs (isoniazid, rifampin, pyrazinamide, and ethambutol hydrochloride) administered for 2 months, followed by 4 months of treatment with isoniazid and rifampin. He denied any history of trauma or sun exposure.

**Figure 1:**
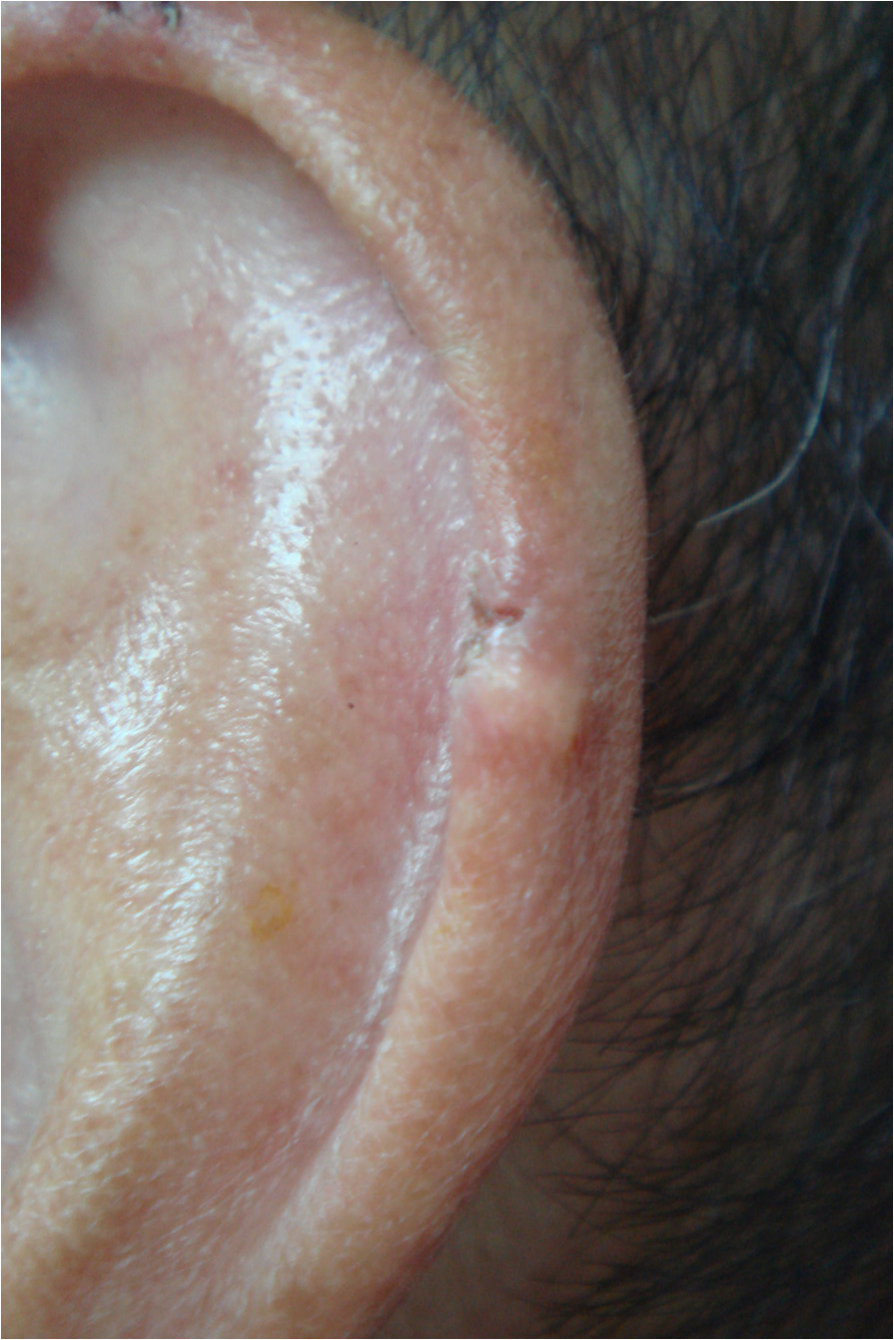
The patient’s left auricle has multiple 2.5-mm firm nodules over the helix.

**Figure 2:**
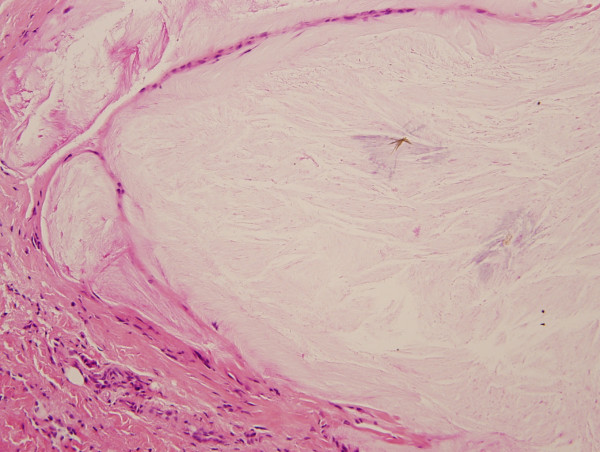
**Hematoxylin-eosin staining of the excised lesion.** The dissolved needle-shaped crystal is surrounded by a proteinaceous matrix on light microscopy.

Physical examination revealed multiple 2.5-mm painless nodules over the helix (Figure 1). These were superficial, circumscribed, and without surrounding erythema or ulceration. An excisional biopsy was performed. The lesion was separated easily from the surrounding tissues and did not invade the perichondrium. Hematoxylin-eosin staining of biopsy specimens revealed amorphous fibrillary crystalline tissue deposits in formalin-fixed tissue, representing a proteinaceous matrix that surrounded dissolved crystals, consistent with gout tophi (Figure 2).

The differential diagnosis of auricular nodules is broad and includes sebaceous cysts, chondrodermatitis nodularis helicis, sarcoid nodules, leprosy, hemangioma, keratoacanthoma, chondroma, lipoma, squamous cell carcinomas, Kaposi’s sarcoma, melanoma, epidermal and dermoid cysts, elastotic nodules, tophaceous gout, rheumatoid nodules, and schwannoma [1]. Biopsy should be performed to rule out malignancy.

Gout is a common systemic metabolic disease, but manifestations of it are infrequently encountered in the field of otolaryngology. It is caused by disordered purine metabolism, resulting in hyperuricemia. When the uric acid level in serum is above 7.0 mg/dL (to convert uric acid level to micromoles per liter, multiply by 59.485), urate crystals form a deposition in subcutaneous tissue, called tophi. Symptoms such as pain, erythema, and swelling are related to the precipitation of uric acid crystals in joint spaces and soft tissues [2]. Primary gout is related to uric acid overproduction, while secondary gout is caused by a decrease in uric acid excretion or by an overproduction of purine. Predisposing factors include aging, male sex, obesity, heavy alcohol consumption, a purine-rich diet, medication use, and genetics. In the patient described herein, the uric acid level was 12.4 mg/dL at 1 week after initiating antituberculosis therapy, and it was 10.1 mg/dL 2 years later. An antituberculosis regimen with pyrazinamide inhibits renal tubular excretion of urate, resulting in some hyperuricemia [3]. Gout tophi occurred at special location like auricular area following a clear history of anti-tuberculosis treatment is unusual. On gross pathological examination, tophaceous gout deposits appear as yellow-white chalky material. The histopathological diagnosis of gouty tophi is made by identifying needle-shaped crystals that are negatively birefringent under polarized light [4]. The deposition of crystals is difficult to visualize with routine fixation, tissue processing, and staining because they are dissolved by formalin-based preservatives. A characteristic feature is a basophilic granular matrix surrounding dissolved crystals on light microscopy.

The external ear, especially the helix, is one of the most common sites of tophus formation in the head and neck region. Documented sites of involvement in the head and neck include the arytenoid, true vocal cord, hyoid bone, thyroid cartilage, nasal septum, temporomandibular joint, soft palate, cervical spine, and glossoepiglottic ligament [5] Tophi of the auricle are usually asymptomatic. Dietary modification and medication use (e.g., a xanthine oxidase inhibitor such as allopurinol or a uricosuric agent such as probenecid) are recommended. Surgical intervention is reserved for tissue diagnosis or for when tophi inflame and ulcerate through overlying skin.

## Conclusions

Tophi of the auricle are usually asymptomatic but can become inflamed and occasionally ulcerate through the overlying skin. Chronic tophaceous gout is treated with dietary control and medication. Surgical excision is performed under local anesthetic if symptoms progression or cosmetically deformity is concerned. These lesions clinically resemble carcinoma and hence even asymptomatic auricular nodules should be biopsied for tissue diagnosis.

## Consent

Written informed consent was obtained from the patient for publication of this case report and any accompanying images. A copy of the written consent is available for review by the Editor-in-Chief of this journal.

## Competing interests

The authors declare that they have no competing interests.

## Authors’ contributions

PCW diagnosed, investigated, followed-up and managed the patient, and determined the medical significance. YCH collected the clinical pictures of the patient. HJC collected the data and wrote the manuscript. PCW and SHH revised the manuscript and provided important suggestions regarding medical content. All authors read and approved the final manuscript.
